# The effects of IL-8, IL- 6, and IL-1 on the risk of celiac disease: a Bayesian regression analysis 

**Published:** 2019

**Authors:** Maryam Nasserinejad, Sadjad Shojaee, Mehdi Ghobakhlou, Elena Lak, Pegah Eslami, Mohamad Amin Pourhoseingholi

**Affiliations:** 1 *Gastroenterology and Liver Diseases Research Center, Research Institute for Gastroenterology and Liver Diseases, Shahid Beheshti University of Medical Sciences, Tehran, Iran*; 2 *Clinical Research Development Unit, Shahid Beheshti Hospital, Hamadan University of Medical Sciences, Hamadan, Iran*; 3 *Basic and Molecular Epidemiology of Gastrointestinal Disorders Research Center, Research Institute for Gastroenterology and Liver Diseases, Shahid Beheshti University of Medical Sciences, Tehran, Iran *

**Keywords:** Celiac disease, Cytokines, Bayesian logistic regression model

## Abstract

**Aim:**

The present study aimed to evaluate the association between serum levels of interleukin IL-1, IL-6, IL-8 genes as well as interferon (IFN)-γ and the risk of celiac disease (CD).

**Background:**

The role of serum cytokine levels in the pathophysiology of CD is still an open field to be explored.

**Methods:**

This case-control study was performed on 110 patients with CD and 46 healthy controls referring to Taleghani Hospital, Tehran, Iran. Expression levels of IL-1, IL-6, IL-8, and IFN-γ were assessed by enzyme-linked immunosorbent assay (ELISA) kits.

**Results:**

The Bayesian intervention odds ratio (OR) and Highest Posterior Density (HPD) interval were 1.133 (95% credible interval 1.018- 1.269), 0.947 (95% credible interval 0.898 - 0.996) and 1.004 (95% credible interval 1.001- 1.009) for IL-1, IL-6, and IL-8 respectively.

**Conclusion:**

The serum level of IFN-γ has no effect on the risk of CD, but given the OR and the HPD interval obtained for serum levels of IL-1, IL-6 and IL-8, with one unit increase in IL-1 serum, the risk of CD grows by 1.13 times while one unit increase in IL-6 serum reduces the risk of CD by 15%. Finally, regarding IL-8, the risk of CD increases by 0.004 times with a unit increase in IL-8 serum.

## Introduction

 Celiac disease (CD) is defined as a gluten-sensitive immune reaction to ingestion of gluten, a protein found in wheat, barley and rye (1). This reaction damages the intestinal mucosal layer and leads to inflammation, which may result in a spectrum of gastrointestinal symptoms, such as diarrhea, fatigue, weight loss, and bloating (2). In addition, it can cause nutritional abnormalities and systemic complications ranging from anemia and osteoporosis to secondary autoimmunity and malignancy (3). The global prevalence of biopsy-confirmed CD has been 0.7% (4), which is 0.4% for the United States (5), 0.8% for Europe and Oceania, and 0.6% for Asia (4, 6). Different studies in Iran have shown that the prevalence of CD is about 1% (7). Note that the prevalence of CD at an early age is higher than that for adults, and is also higher in women than in men (4). 

CD arises in genetically susceptible individuals with HLA-DQ2 or HLA-DQ8 alleles and is characterized by a T-cell-driven inflammation in the proximal small bowel triggered by gluten ingestion (8). Gluten is delivered to TCD4+ cell and activates them causing the secretion of different pro-inflammatory and inflammatory cytokines such as interferon (IFN)-γ, interleukin IL-1, IL-6, IL-8, IL-15, IL-18, IL17, and IL-21 genes (9, 10). After this event, TCD4+ cell interaction with B cells leads to the release of autoantibodies such as anti-tissue transglutaminase (tTG) and DGA (Deamidated gliadin antibody) which is a marker for active disease (11). 

Several studies have found increased levels of IL-2, IL-4, IL12, IL-10, and IFN-γ in patients with CD as compared with healthy population (12-16). Cytokines are implicated in both enhancing and suppressing immune responses through their influence on T-cells and other immune effectors. IL-2, IL-12, and INF-γ activate T helper type 1 (Th-1) lymphocytes (17), while IL-4, IL-5, and IL-10 lead to activation of T helper type 2 (Th-2) cell (18). IL-8 is a chemokine which is expressed in tissues due to infiltration of neutrophils and plays an important role in immune responses due to neutrophils. In addition, IL-6 gene is secreted from various immune cells. Based on few studies, the levels of IL-6 and IL-8 have been shown to be higher in comparison with the healthy group (19-21). 

Considering previous studies on the role of interferon and interleukin genes in the pathogenesis of CD disease and the lack of information about the expression levels of these biomarkers in Iranian celiac patients, we evaluated the effects of serum cytokine levels of IL-1, IL-6, IL-8, and IFN-γ in individuals with CD as compared to healthy individuals as the control group.

One of the factors that directly affects the accuracy of the results of a study is the sample size (22). It has also been proved that inference on large data sets can be highly inaccurate if applied to logistic regression with small samples as the sample size has the greatest impact on the performance of the logistic model predictions (23, 24). Indeed, when the sample size is small or medium, the results of logistic regression are not reliable (25). As a result, Bayesian methods can be used under such circumstances, as it has been proved that Bayesian estimates based on 25% samples have predictive validity approximately equal to that of classic estimates (maximum likelihood estimates) based on complete samples .(26) In addition, the simulation results have demonstrated that Bayesian estimations provide more stable distributions (27). 

In this study, since the sample size has not been large and several predictor variables have been examined, we decided to use Bayesian methods to avoid inaccurate results. 

## Methods

**Study design**

We used the data collected in the research institute for Gastroenterology and liver diseases, Tehran, Iran in 2016. The data included 110 patients with celiac disease and 46 healthy individuals, In addition to demographic information, serum levels of IL-1, IL-6, IL-8, and IFN-γ were also measured via commercial ELISA kits, according to the manufacturer’s instructions (28)**.**

**Bayesian Logistic regression model**

Initially, a probability model must be selected for the available data. In this study, Bernoulli distribution model was appropriate. In addition, it is necessary to select a prior distribution. Then, to designate the posterior distribution, the prior distribution is multiplied by the likelihood function. Lastly, the posterior distribution is estimated by Markov Chain Monte Carlo (MCMC) (29). We had no prior knowledge for the parameters. Therefore, we used a non–informative prior on the parameters. We chose a weakly informative normal prior distribution with location parameter 0 and scale parameter 1.5 to assign equal probabilities to all possibilities. The open source statistical software R version 3.6.0 and packages coda and rjags were used for all analyses.

**Markov Chain Monte Carlo method and Metropolis**

It is difficult to calculate the posterior distribution due to the complexity of the integrals, as application of direct techniques becomes challenging. Thus, MCMC methods are proposed (30). MCMC methods simulate values of random variables from the posterior distribution. Indeed, a Markov chain is constructed, where the most important property of this chain is allowing the next value of each parameter vector to depend on the current value but not on the previous one. The second important property is that the simulation algorithm is repeated multiple times and as a result the approximation of the posterior distribution improves at every step. Thus, the posterior distributions can be approximated with high accuracy. A major consideration in MCMC simulations is that of convergence; we used Gelman-Rubin diagnostic for this issue and autocorrelation function for the estimated autocorrelation for each node. 

## Results

In this study, there were 110 (70.5%) patients with CD as case and 46 (29.5%) controls. The mean SD of age was 33.56 13.76 years in the case group and 38.78 11.15 years in the control group which is a significant difference (P=0.02). In the case group, there were 38 males and 72 females while in the control group there were 19 males and 27 females; according to the results of Chi-square test, there was no significant difference in sex (P=0.42). According to the results of Bayesian logistic regression, serum levels of IL-1, IL-6, and IL-8 had a significant effect on CD but no significant relationship was found between interferon (IFN)-γ and CD. The Bayesian intervention odds ratio (OR) and highest posterior density interval (HPD) were 1.133 (95% credible interval 1.018- 1.269), 0.947 (95% credible interval 0.898 - 0.996), and 1.004 (95% credible interval 1.001- 1.009) for IL-1, IL-6, and IL-8, respectively (Table 1). 

**Table 1:** The results of the Bayesian logistic regression

Variable	OR	95% HPD interval
IL-1	1.133	1.018- 1.269*
IL-6	0.947	0.898 - 0.996*
IL-8	1.004	1.001- 1.009*
IFN-γ	1.003	0.999- 1.007

**Figure 1:**
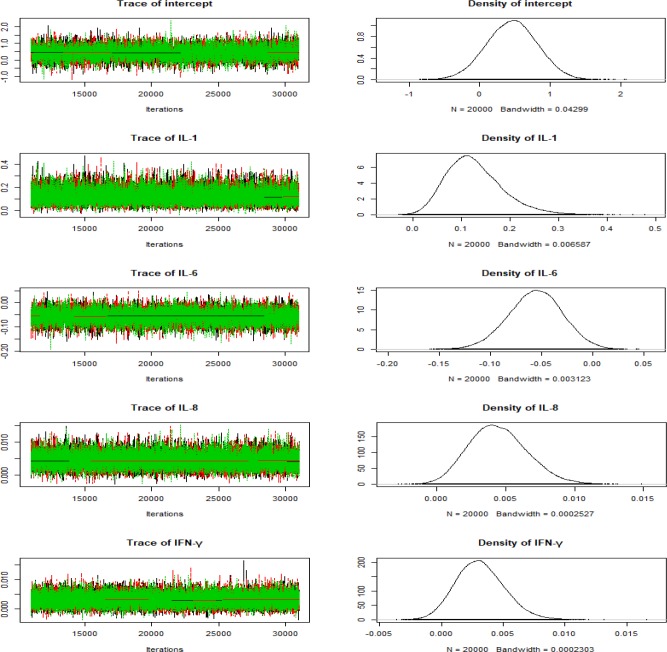
Histories of Markov chain, Trace and Density plots of each variable

In addition, according to the diagram, the posterior distribution of the parameters is approximately normal. Also, as can be seen, the trace plots do not have strong fluctuations, indicating that the Bayesian model is convergent. Thus, the results are definitely reliable (Figure 1).

## Discussion

Celiac disease is an autoimmune disease that has been studied extensively to determine its causes and symptoms. So far, many biomarkers that have been shown to affect celiac have been proven. In this study, we worked on the impact of IL-1, Il-6, IL-8, and IFN-γ on the risk of CD. According to our results, the serum level of IFN-γ had no effect on the risk of CD, but given the OR and the HPD interval obtained for serum levels of IL-1, IL-6 and IL-8, one unit increase in IL-1 serum can raise the risk of CD by 1.13 times while one unit increase in IL-6 serum reduces the risk of CD by 15%. Further, the risk of CD increases by 0.004 times with a unit increase in IL-8 serum. 

The IL-1 family consists of 11 cytokines and has been also expanded to 9 distinct genes that have emerged as a therapeutic mark for auto-inflammatory diseases such as CD. In addition, it has been demonstrated that the IL-1 ligand is more associated with acute and chronic inflammation than any other cytokine family (31). IL-1α and IL-1β have highly inflammatory effects, and have been evaluated in various studies. Fornari et al. (32) showed that elevated serum IL-1 and IL-1β was associated with increased CD and its symptoms, which was consistent with the results of the present study. 

IL-8 is known as a potent promoter of angiogenesis and neutrophil chemotactic factor. It produces chemotaxis in target cells, at first neutrophils and then other granulocytes, causing them to migrate toward the site of infection. Note that it is also a member of the CXC chemokine family (33). IL-6 is an interleukin that acts as a pro-inflammatory cytokine as well as an anti-inflammatory myokine with extensive functions (34). Many studies have considered that IL-8 and IL-6 are influential factors in the risk of CD, which is completely consistent with our results (35-39).

IFN-γ (type II interferon) is a cytokine which is critical for innate and adaptive immunity against viral as well as some bacterial and protozoal infections (40). According to the results of the Lopez-Palacios et al. (41) study, IFN-γ rises in CD group as compared with the control group. In addition, a study by Marafini et al. (42) on mice showed that TNF-α and IFN-γ were more abundant in active CD mucosa compared to controls (42). In our study, the results also showed that an increase in IFN-γ results in elevated risk of CD, but its value to be considered as a risk of CD was very low and not significant.

In conclusion, the serum level of IFN-γ has no effect on the risk of CD and its symptoms. However, given the OR and the HPD interval obtained for serum levels of IL-1, IL-6, and IL-8, with one unit increase in IL-1 serum, the risk of CD grows by 1.13 times while one unit increase in IL-6 serum reduces risk of CD by 15%. Finally, regarding IL-8, the risk of CD increases by 0.004 times with a unit increase in IL-8 serum.

## References

[1] Ludvigsson JF, Leffler DA, Bai JC, Biagi F, Fasano A, Green PH (2013). The Oslo definitions for coeliac disease and related terms. Gut.

[2] Rostami-Nejad M, Hogg-Kollars S, Ishaq S, Rostami K (2011). Subclinical celiac disease and gluten sensitivity. Gastroenterol Hepatol Bed Bench.

[3] Khoshbaten M, Rostami Nejad M, Farzady L, Sharifi N, Hashemi SH, Rostami K (2011). Fertility disorder associated with celiac disease in males and females: fact or fiction?. J Obstet Gynaecol Res..

[4] Singh P, Arora A, Strand TA, Leffler DA, Catassi C, Green PH (2018). Global prevalence of celiac disease: systematic review and meta-analysis. Clin Gastroenterol Hepatol.

[5] Fasano A, Berti I, Gerarduzzi T, Not T, Colletti RB, Drago S (2003). Prevalence of celiac disease in at-risk and not-at-risk groups in the United States: a large multicenter study. Arch Intern Med.

[6] Ashtari S, Pourhoseingholi MA, Rostami K, Aghdaei HA, Rostami-Nejad M, Busani L (2019). Prevalence of gluten-related disorders in Asia-Pacific region: a systematic review. J Gastrointestin Liver Dis.

[7] Ahadi Z, Shafiee G, Razmandeh R, Keshtkar AA, Sani MN, Azemati B (2016). Prevalence of celiac disease among the Iranian population: A systematic review and meta-analysis of observational studies. Turk J Gastroenterol.

[8] Dieterich W, Ehnis T, Bauer M, Donner P, Volta U, Riecken EO (1997). Identification of tissue transglutaminase as the autoantigen of celiac disease. Nat Med.

[9] Maiuri L, Ciacci C, Ricciardelli I, Vacca L, Raia V, Auricchio S (2003). Association between innate response to gliadin and activation of pathogenic T cells in coeliac disease. Lancet.

[10] Faghih M, Rostami-Nejad M, Amani D, Sadeghi A, Pourhoseingholi MA, Masotti A (2018). Analysis of IL17A and IL21 Expression in the Small Intestine of Celiac Disease Patients and Correlation with Circulating Thioredoxin Level. Genet Test Mol Biomarkers.

[11] du Pre MF, Sollid LM (2015). T-cell and B-cell immunity in celiac disease. Best Pract Res Clin Gastroenterol.

[12] Bjorck S, Lindehammer SR, Fex M, Agardh D (2015). Serum cytokine pattern in young children with screening detected coeliac disease. Clin Exp Immunol.

[13] Ma X, Yan W, Zheng H, Du Q, Zhang L, Ban Y (2015). Regulation of IL-10 and IL-12 production and function in macrophages and dendritic cells. Res.

[14] Mosser DM, Zhang X (2008). Interleukin-10: new perspectives on an old cytokine. Immunol Rev.

[15] Liu J, Cao S, Kim S, Chung EY, Homma Y, Guan X (2005). Interleukin-12: an update on its immunological activities, signaling and regulation of gene expression. Curr Immunol Rev.

[16] Viallard JF, Pellegrin JL, Ranchin V, Schaeverbeke T, Dehais J, Longy-Boursier M (1999). Th1 (IL-2, interferon-gamma (IFN-gamma)) and Th2 (IL-10, IL-4) cytokine production by peripheral blood mononuclear cells (PBMC) from patients with systemic lupus erythematosus (SLE). Clin Exp Immunol.

[17] Weiss JM, Subleski JJ, Wigginton JM, Wiltrout RH (2007). Immunotherapy of cancer by IL-12-based cytokine combinations. Expert Opin Biol Ther.

[18] Iyer SS, Cheng G (2012). Role of interleukin 10 transcriptional regulation in inflammation and autoimmune disease. Crit Rev Immunol.

[19] Wong R, Wilson R, Steele R, Radford-Smith G, Adelstein S (2002). A comparison of 13 guinea pig and human anti-tissue transglutaminase antibody ELISA kits. J Clin Pathol.

[20] Shan L, Molberg Ø, Parrot I, Hausch F, Filiz F, Gray GM (2002). Structural basis for gluten intolerance in celiac sprue. Science.

[21] Abadie V, Sollid LM, Barreiro LB, Jabri B (2011). Integration of genetic and immunological insights into a model of celiac disease pathogenesis. Annu Rev Immunol.

[22] Claeskens G, Aerts M, Molenberghs G (2003). A quadratic bootstrap method and improved estimation in logistic regression. Statistics & probability letters.

[23] Potter DM (2005). A permutation test for inference in logistic regression with small‐and moderate‐sized data sets. Stat Med.

[24] Pearce J, Ferrier S (2000). An evaluation of alternative algorithms for fitting species distribution models using logistic regression. Ecol Modell.

[25] Maiti T, Pradhan V (2009). Bias reduction and a solution for separation of logistic regression with missing covariates. Biometrics.

[26] Schulz EM, Betebenner D, Ahn M (2004). Hierarchical logistic regression in course placement. J Educ Meas.

[27] Gordóvil Merino A, Guàrdia Olmos J, Peró M (2012). Estimation of logistic regression models in small samples A simulation study using a weakly informative default prior distribution. Psicologica.

[28] Heydari F, Rostami-Nejad M, Moheb-Alian A, Mollahoseini MH, Rostami K, Pourhoseingholi MA (2018). Serum cytokines profile in treated celiac disease compared with non-celiac gluten sensitivity and control: a marker for differentiation. J Gastrointestin Liver Dis.

[29] Glickman ME, Van Dyk DA (2007). Basic Bayesian methods. Topics in biostatistics.

[30] Gamerman D, Lopes HF (2006). Markov chain Monte Carlo: stochastic simulation for Bayesian inference.

[31] Dinarello CA (2011). Interleukin-1 in the pathogenesis and treatment of inflammatory diseases. Blood.

[32] Fornari MC, Pedreira S, Niveloni S, Gonzalez D, Diez RA, Vazquez H (1998). Pre- and post-treatment serum levels of cytokines IL-1beta IL-6 and IL-1 receptor antagonist in celiac disease Are they related to the associated osteopenia?. Am J Gastroenterol.

[33] Seppola M, Larsen AN, Steiro K, Robertsen B, Jensen I (2008). Characterisation and expression analysis of the interleukin genes, IL-1β, IL-8 and IL-10, in Atlantic cod (Gadus morhua L). Mol Immunol.

[34] Ferguson-Smith AC, Chen YF, Newman MS, May LT, Sehgal PB, Ruddle FH (1988). Regional localization of the interferon-beta 2/B-cell stimulatory factor 2/hepatocyte stimulating factor gene to human chromosome 7p15-p21. Genomics.

[35] Di Sabatino A, Giuffrida P, Fornasa G, Salvatore C, Vanoli A, Naviglio S (2016). Innate and adaptive immunity in self-reported nonceliac gluten sensitivity versus celiac disease. Dig Liver Dis.

[36] Goel G, Tye-Din JA, Qiao SW, Russell AK, Mayassi T, Ciszewski C (2019). Cytokine release and gastrointestinal symptoms after gluten challenge in celiac disease. Sci Adv.

[37] Vorobjova T, Tagoma A, Oras A, Alnek K, Kisand K, Talja I (2019). Celiac disease in children, particularly with accompanying type 1 diabetes, is characterized by substantial changes in the blood cytokine balance, which may reflect inflammatory processes in the small intestinal mucosa. J Immunol Res.

[38] Bjorck S, Brundin C, Karlsson M, Agardh D (2017). Reduced Bone Mineral Density in Children With Screening-detected Celiac Disease. J Pediatr Gastroenterol Nutr.

[39] Kordulewska NK, Kostyra E, Piskorz-Ogorek K, Moszynska M, Cieslinska A, Fiedorowicz E (2019). Serum cytokine levels in children with spectrum autism disorder: Differences in pro- and anti-inflammatory balance. J Neuroimmunol.

[40] Schoenborn JR, Wilson CB (2007). Regulation of interferon‐γ during innate and adaptive immune responses. Adv Immunol.

[41] Lopez-Palacios N, Pascual V, Castano M, Bodas A, Fernandez-Prieto M, Espino-Paisan L (2018). Evaluation of T cells in blood after a short gluten challenge for coeliac disease diagnosis. Dig Liver Dis.

[42] Marafini I, Monteleone I, Di Fusco D, Cupi ML, Paoluzi OA, Colantoni A (2015). TNF-alpha producing innate lymphoid cells (ilcs) are increased in active celiac disease and contribute to promote intestinal atrophy in mice. PLoS One.

